# Young women at risk: Harassment by gender, race, and age in the gig economy

**DOI:** 10.1371/journal.pone.0359956

**Published:** 2026-11-04

**Authors:** Julieta Aguilar, Jeremy Reynolds

**Affiliations:** Department of Sociology, Purdue University, West Lafayette, Indiana, United States of America; UMass Amherst: University of Massachusetts Amherst, UNITED STATES OF AMERICA

## Abstract

Harassment at work is a serious issue, and gig workers often lack protections that shield conventional employees from mistreatment. Few studies, however, have examined harassment in gig work quantitatively. Guided by the intersectional perspective and the perpetrator-predation framework, we use 2021 data from Pew’s American Trends Panel to examine harassment among gig workers at the intersections of race, gender, and age. We find that Black and Hispanic workers report more harassment than White workers regardless of gender or age. Furthermore, although women do not report more harassment than men on average, the connection between gender and harassment is strong among young workers: young women report notably more harassment than young men and more harassment than any other gender-age group. Consequently, harassment is highest for young women, especially those who are Black or Hispanic. For young women, gig work is thus both economically precarious and associated with a heightened risk of harassment.

## Introduction

The growth of the gig economy has led to considerable research, and that research highlights a divide between gig work and regular jobs in the conventional economy. By gig work, we mean work performed by people in the formal economy who use apps or websites of intermediaries to interact with customers. This includes work on platforms like Uber, DoorDash, and Airbnb, but not sex work, day labor, music “gigs,” or other off-the-books work in the informal, underground economy.

Unlike conventional employees, gig workers do not typically have supervisors, and they are usually classified as independent contractors rather than employees [1]. Consequently, platforms like Uber, DoorDash, and TaskRabbit are able to avoid most responsibilities associated with being an employer, like providing a minimum wage or meeting safety standards [1,2]. Nevertheless, many people still see gig work as a useful source of extra income and value the ability to set their own schedules and be their own boss [3,4]. Scholars and worker advocates, however, often emphasize that gig work involves risks because it lacks many protections that most conventional jobs provide [1,5,6].

Few studies, however, have examined the risk of harassment in the gig economy. This topic deserves more attention because harassment is harmful to workers and organizations [7–9] and because gig workers typically lack workplace and legal protections that shield conventional workers from harassment [1,10,11]. Furthermore, the handful of studies that have examined harassment in the gig economy suggests that it is a common problem [10,12–14]. Moreover, given the frequency of contact between gig workers and customers, instances of harassment could be quite numerous. In the fourth quarter of 2024, Uber drivers alone completed roughly 33 million trips per day [15]. The number of worker-customer interactions across the entire gig economy over the course of one year is thus enormous. Currently, we know very little about the extent to which those interactions expose gig workers to harassment.

In this paper, we extend the small literature on harassment of gig workers by discussing how the nature of gig work shapes the context for harassment and by estimating how the frequency of harassment reflects the intersection of race, gender, and age. Our analysis draws on a national sample of gig workers from Wave 94 of the American Trends Panel gathered by the Pew Research Center in August of 2021. Our results provide the most complete and nuanced quantitative picture of harassment in the gig economy to date. Our analysis also answers repeated calls for more research that examines harassment using an intersectional perspective [7,16–18] and research that examines harassment in precarious work [7,19–21].

## Background

### Defining harassment at work

The prevalence of harassment at work is difficult to assess because there is no agreed-upon definition of harassment. In fact, there is not even a “gold standard” definition of *sexual* harassment [16]. The *legal* definition of sexual harassment, for example, imposes statutes of limitations and other requirements that greatly restrict what counts as sexual harassment in court [16]. Due to these restrictions and the difficulty of reporting, the number of sexual harassment cases filed with the Equal Opportunity Employment Commission (EEOC) massively undercounts sexual harassment incidents at work [22]. To provide a more comprehensive assessment, many researchers thus rely on survey data (rather than EEOC filings) and use a broad definition of sexual harassment that includes 1) sexual coercion 2) unwanted sexual attention and 3) gender harassment [7,16]. Including gender harassment expands the definition substantially to encompass behavior that does not aim to elicit sexual cooperation. Gender harassment is an expression of contempt that often involves belittling, isolating, or threatening people because of their gender [7].

For assessing harassment of gig workers, we use a broad definition that includes both sexual harassment and other types of harassment. Our approach is thus similar to (but somewhat narrower) than the concept of “workplace mistreatment,” which includes “all forms of interpersonal behaviors that employees can engage in or experience that are negative or harmful to the target” [23]. We use the term harassment rather than mistreatment because we do not examine mistreatment that can occur without personal interactions, e.g., wage discrimination and ostracism [8,20]. We also prefer the term harassment over incivility because the latter focuses exclusively on low-intensity mistreatment [24]. Studying harassment helps us capture a range of interpersonal interactions that are harmful, may or may not be repetitive or obviously directed at a protected group, can range from low to high intensity, and can be legal or illegal.

Although it can be useful for legal or theoretical reasons to differentiate between harassment motivated by particular characteristics (race, gender, age, etc.), we do not focus on such distinctions. Harassment based on race, gender, and age are often intertwined [16,25]. When a Black woman is harassed, for instance, it may be sexual harassment, racial harassment or both. The same complication exists for other combinations of race and gender. When Black men are harassed, it can also reflect their race, their gender, or their distinct location at the intersection of race and gender. Each group can face different stereotypes and be harassed for different reasons, and they may thus have different risks of harassment. Black women, for instance, are often stereotyped as more promiscuous than White women, and Black men are more likely than White men to be stereotyped as sexual predators [26]. Furthermore, although Black men are likely to be harassed for deviating from heterosexual masculinity norms, Black women may be harassed for deviating from heterosexual femininity norms [7]. Sexual stereotypes and their consequences thus vary by race, and racial stereotypes and their consequences vary by gender. Using a broad definition of harassment helps us examine the combined effect of race and gender and account for the insight that individuals always embody multiple identities. Empirical patterns reflect this complexity. For instance, between 2018 and 2021, 71.2% of workplace sexual harassment charges filed by African Americans were filed concurrently with race-based harassment charges [27].

### Patterns of harassment by race, gender, and age

Studies using similar broad definitions have documented substantial harassment in the conventional economy, and they often find that it is patterned by race, gender, and age. Despite variations in how it is defined and measured, harassment at work is a common occurrence worldwide [28]. In 2023, roughly 23% of employed adults in the United States said they were harassed at work in the last 12 months [29]. The risk of harassment among conventional employees is especially high for workers of color, women, and older workers [30]. In 2020, roughly 33% of all work related harassment charges filed with the EEOC were based on race, 32% were based on sex, and 21% were based on age; disability is the only other personal characteristic that motivates many EEOC filings [31]. Analyses of the General Social Survey also suggest that workplace harassment is often based on race, sex, or age [25,32]. These results are consistent with the perpetrator-predation paradigm, which suggests that lower-status people are more likely to be harassed because they are seen as easier targets [24,30,33].

Recent meta-analyses of “workplace mistreatment” and “workplace incivility,” however, have found mixed evidence of demographic differences. One meta-analysis found that women and men report similar levels of mistreatment in general (they differ only in perceptions of gender-based mistreatment) and that White respondents and people of color report similar levels of mistreatment (except when reporting race-based mistreatment) [17]. A second meta-analysis found that reports of workplace incivility are similar for men and women but higher among people of color than among White workers [33]. A third meta-analysis found that workplace incivility was higher for women and people of color [24]. Conclusions regarding age are also mixed. One meta-analysis found very weak evidence of age differences in mistreatment [17], but others find higher levels of mistreatment among younger workers [33,34]. These mixed findings are rather surprising because theory and prevailing wisdom suggest that demographic differences in harassment (especially by gender and race) will be substantial [17].

There is stronger evidence for the perpetrator-predation paradigm’s suggestion that harassment reflects workplace context [17,24,33]. Harassment is more common in workplaces where leaders are reluctant to punish problematic behaviors or where policies and practices do not convey an expectation that civility norms will be enforced [33]. According to the logic of the perpetrator-predation paradigm, this is because perpetrators are more likely to engage in harassment in contexts where they think they can avoid punishment [24].

We examine variations in harassment by gender, race, and age in the gig economy because we suspect that the demographic composition of the gig workforce and the lack of worker protections may exacerbate group differences. We reason that because gig work provides few protections from harassment, perpetrators will be emboldened to seek targets who also appear vulnerable because of their demographics, thus generating group differences. Below we describe gig work, how the nature of that work may influence patterns of harassment, and the handful of studies that have examined harassment of gig workers.

### Defining gig work

Definitions of gig work vary, but it is often defined as work that involves short-term engagements among workers and customers in the formal economy that are facilitated by an online platform that people access through a computer or app [2,35]. For these reasons, it is also sometimes called platform work [2]. The prevalence of gig work is difficult to assess because most large U.S. labor force surveys do not measure it well [36,37], and because private studies focused on gig work use a variety of definitions and measurement strategies [38]. Still, some estimates indicate that roughly 4.5% of U.S. adults earned income through a gig platform in 2018 [39] and 16% had done some gig work by 2021 [14].

Gig work is also a type of precarious work. It generally lacks predictable income or work hours, fringe benefits, and the legal protections that conventional employment provides [1,11,40]. Despite these shortcomings, gig work provides earning opportunities that are not available in the conventional labor market [41]. Consequently, workers who need the income may do gig work despite its objectively “bad” characteristics [42,43], including the mental distress it can cause [44,45]. These characteristics may create a work context that both attracts vulnerable workers to gig work and allows harassment to go unchecked [7,19,21].

Apart from the similarities described above, gig work is heterogeneous. It can entail driving customers from location to location (Uber & Lyft), delivering food (DoorDash & Instacart), renting personal properties (Airbnb), or completing general or specialized online tasks (Amazon Mechanical Turk & Fiverr). Each type of gig work differs in its requirements, compensation, and risks [46]. For example, to earn money through ride-hailing apps, workers must own a vehicle. Other types of gig work, in contrast, only require an electronic device for doing online work. Furthermore, because there is no regulation of pay in the gig economy, earnings differ substantially between platforms [1]. On average, platforms that require workers to have more wealth and assets also tend to pay more [47]. Gig work that requires more expertise also tends to pay better [48]. Finally, although many types of gig work require close contact with customers, some gig work allows people to work remotely – often from home (e.g., Amazon Mechanical Turk, Upwork, etc.). Remote gig work seems to offer substantial advantages over in-person gig work [46], but whether the advantages include protection from harassment is unclear.

### Gig work and harassment

Harassment in the gig economy may be quite different from harassment in conventional jobs for a variety of reasons. Gig work tends to attract specific types of workers, who may be more vulnerable to harassment than conventional workers. Gig work and conventional work also tend to occur in different locations, involve different social interactions, and offer different protections from harassment. Below, we explain briefly why these differences may be important.

Gig workers are demographically different from people who do not do gig work, and they are probably different from workers with conventional jobs. National surveys of gig workers in the U.S. are rare, but reports from the Pew Research Center provide some of the most comprehensive demographic comparisons of people who have done gig work and people who have not [14]. According to the Pew Research Center, Hispanic, Black, and Asian adults in the United States are much more likely than White adults to do gig work, and young people are much more likely than older people to do gig work. Women, in contrast, are only slightly more likely than men to have some experience with gig work. Comparing these patterns to labor force participation rates in the conventional economy suggests indirectly that gig workers are different from workers with conventional jobs. Labor force participation rates in the conventional economy are similar across racial groups, they are low among young people, and they are substantially higher among men than among women [49]. The gig workforce thus seems to be more non-white, young, and female than the conventional labor force. If those characteristics are associated with harassment, gig workers might report more harassment than conventional workers due to demographic differences. Indeed, the perpetrator-predation framework suggests that lower-status groups (like non-white and female workers) may face a higher risk of harassment [33].

Meta-analyses of workplace mistreatment suggest that the context of work shapes the risk of harassment even more than demographics [24,33], and gig work has a number of characteristics that may allow harassment to flourish. Gig work often occurs in different locations than conventional work and involves different social interactions. First, gig workers do not usually work with people who might protect them. For conventional employees, co-workers are sometimes the perpetrators of harassment [50], but co-workers and supervisors are also often an important sources of protection [51]. Gig workers, however, do not typically have co-workers or supervisors. The risk of harassment may thus stem primarily from interactions with customers and resemble patterns of customer incivility or aggression [52]. Second, perpetrators may target gig workers because of the incentives that encourage gig workers to tolerate harassment. In conventional service jobs, workers who depend on tips are often targeted by perpetrators who know that the fear of losing a tip can encourage workers to overlook bad behavior [34]. Gig workers face similar pressures: they depend on tips and customer ratings, and they may need to engage in carefully orchestrated behaviors to deviate from customer expectations without being punished [53]. Maffie argues that by incorporating these power inequalities into their apps, gig platforms “launder control through customers” and expose workers to customer abuse [54]. Third, in contrast to many service workers, gig workers do not typically work in traditional “workplaces.” Much of their work is done where customers are located, without onlookers who might deter harassment. Gig workers are often alone with customers in cars and houses and as they travel to meet customers [10,13,55]. Patterns of gig worker harassment might thus resemble patterns of harassment outside workplaces. Notably, more sexual harassment takes place in public than in workplaces, and when harassment is examined both inside and outside work, women report more of it than men [50]. The frequent interactions with customers and the solitary nature of the work may heighten gig workers’ vulnerability to perpetrators who are inclined to harass vulnerable, isolated targets.

The harassment of gig workers may also reflect their lack of organizational and legal protection. Gig workers are not protected by laws that prohibit harassment or discrimination at work [10,11]. Also, they often lack access to workplace supports that could help prevent harassment or facilitate the reporting of complaints (e.g., HR professionals, protective supervisors or co-workers etc.). This lack of protection makes them vulnerable targets [8]. Even when gig platforms provide ways to report harassment [as with Uber, see 10], workers who report harassment may still face a substantial risk of retaliation. Of all the workplace harassment charges filed in the United States in 2020, over half were about retaliation [31]. Furthermore, in contrast to conventional workers, gig workers are not protected from retaliation. Customers can retaliate by leaving bad reviews, filing a complaint, or by getting verbally or physically aggressive [52]. Gig workers are also vulnerable to retaliation from gig platforms themselves, which can deactivate workers for a wide variety of reasons [12]. Several authors have suggested that in precarious work like gig work, this transfer of legal and organizational responsibility for safety from employers to workers may contribute to harassment [7,19–21].

To date, although there is a large literature on gig work and well-being [56], very few studies examine gig workers’ experiences with harassment. The handful of studies on the topic, however, provides important information. They clarify why gig workers are so vulnerable to harassment and the strategies they use to protect themselves. They also begin to describe the prevalence and patterns of harassment.

Existing qualitative studies explain why gig workers are so vulnerable and how they try to protect themselves. A study of thirty-two ride-share drivers (mostly men) revealed that they perform “defensive labour” by regulating their emotional responses to harassment (e.g., repressing anger, acting tough, maintaining rapport) while simultaneously trying to identify the best way to avoid harm [12]. Other qualitative studies show that drivers may respond to harassment by giving customers low ratings, ending a ride early, fleeing the vehicle, responding with verbal or physical aggression, carrying mace or weapons, or calling the police [10,12]. A study of ride-share drivers, food delivery workers, and home service gig workers, however, found that because confronting passengers in cars and private places is risky and can reduce a worker’s earnings potential, some women drivers “brush off” harassment to avoid both escalation and diminished access to work [13].

The only quantitative analysis of harassment among gig workers that we have found is embedded in a more general study of U.S. gig workers and their experiences [14]. The study, which was conducted by the Pew Research Center, indicates that harassment is common, and the report highlights three different forms of harassment. About 37% of gig workers report that they are sometimes or often treated rudely by customers. Around 35% have sometimes or often felt unsafe. Roughly 19% say they sometimes or often experience unwanted sexual advances.

The study also highlights some demographic differences in those types of harassment. Unwanted sexual advances are reported more often by workers of color, women, and young workers (than by White, male, or older workers respectively). Feeling unsafe is a more frequent problem for workers of color and young workers, but there is no significant gender difference. Young workers are more likely than older workers to report rude treatment, but differences by race and gender are not significant.

### Hypotheses about race, gender, and age

We extend previous research, in part, by examining how overall levels of harassment vary by race, gender, and age. Examining overall levels of harassment is important for forming an assessment of the risks gig workers face. Specific demographic groups might report different kinds of harassment but similar overall levels. Alternatively, there may be group differences in overall harassment that are only evident when individual kinds of harassment are considered together. Although meta-analyses have found mixed evidence of demographic differences in harassment among conventional workers [33], the perpetrator-predation paradigm suggests that people of lower social status are more likely to be harassed [24,30]. Also, we suspect that group differences may be pronounced in work environments that offer few protections. Therefore, we make the following hypothesis about harassment in gig work:

H1: People of color, women, and young workers will experience more harassment than workers who are white, men, and older.

### Hypotheses about intersectionality

We also extend previous research by examining harassment from an intersectional perspective. The intersectional perspective suggests that the experiences of individuals reflect the combined influence of all the identities they hold simultaneously, and that single identities like gender and race cannot be fully understood in isolation [57–59]. The theory has been widely used to examine how various outcomes reflect the combined effects of race, gender, and other social identities [59] including age [60]. Crenshaw was one of the first to formalize this perspective in an effort to explain workplace discrimination against Black women [61].

Scholars have argued explicitly that researchers should use an intersectional perspective to study workplace harassment [7,16–18], and although there has been some increased attention to this idea, recent reviews of research on sexual harassment and incivility hardly mention intersectionality [20,24,33,62]. One analysis of existing qualitative studies used an intersectional framework and concluded that conventional workers who identify with multiple minority groups experience unique forms of microaggressions [63]. Also, a study using data from the General Social Survey concluded that workplace discrimination reflects the combined influence of gender and age such that women experience persistently high discrimination across the life course while men experience less mistreatment with age [25]. Finally, analyses of EEOC data using an intersectional perspective indicate that between 1996 and 2016, sexual harassment changed in very different ways for Black and White women [64].

We did not find any studies examining harassment among gig workers using an intersectional approach, and it is important to address this gap in the literature for theoretical and empirical reasons. If people experience the combined effect of their identities simultaneously, it may not be possible to explain those experiences well using theoretical models which assume that race, gender, and other factors operate independently. Accounting for intersectionality is also important for empirical accuracy. If the relationship between an identity (e.g., gender) and an experience (e.g., harassment) can vary substantially depending on the other characteristics a person has (e.g., their age), analyses that ignore that variation and focus instead on averages will be overly simplistic or misleading. It is possible, for instance, that mixed results regarding the connection between harassment and race, gender, and age have overlooked significant patterns that reflect the intersections of those identities. Motivated by these considerations, we present an “intercategorical” intersectional analysis [59], i.e., one that seeks to identify which intersections matter for harassment in the context of gig work.

Because the intersectional approach encourages attention to the *potential* ramifications of intertwined identities, we offer hypotheses that foreground that idea. Like others, we predict that women of color will face a “double jeopardy” (i.e., an especially high risk of harassment) because they occupy two disadvantaged social positions simultaneously [7,16–18,65]. Some studies of harassment among conventional workers do not find this type of moderating relationship where the combined effect of gender and race is greater than the sum of their separate effects [65,66]. Nevertheless, we suspect that women of color might experience more harassment than other race-gender groups in the context of gig work because protections are so low. Indeed, intersectionality emphasizes that the outcomes associated with any particular intersection of identities may vary from one context to another [59]. We thus offer a hypothesis about race and gender in gig work that highlights the spirit of the intersectional perspective:

H2: Black and Hispanic women will report more harassment than other race-gender groups.

The same logic suggests that young women will experience comparatively high levels of harassment. Young employees experience much mistreatment in workplaces in general [33] and by customers in particular [34]. The Pew report [14] finds a similar pattern: compared to older workers, young gig workers report more unwanted sexual advances and rude treatment and are more likely to feel unsafe. Research by Ma [13] argues that gender is also an important determinant of harassment in the gig economy. Finally, there is some direct evidence that age and gender are intertwined, at least in the conventional economy: young women bear a double burden of age-based and gender-based mistreatment at work [25]. Based on this evidence and the logic of the intersectional approach, we also hypothesize distinct outcomes for young women in gig work:

H3: Young women will report more harassment than other gender-age groups.

Finally, there appears to be very little research examining how harassment at work reflects the intersection of race and age. Nevertheless, existing research suggests that people of color and younger workers experience some types of harassment more often than White and older gig workers [14]. Based on this information and the spirit of the intersectional perspective, we offer one final hypothesis:

H4: Young Black and Hispanic gig workers will report more harassment than other race-age groups.

## Methods

### Data

To test these hypotheses, we draw on the Pew Research data used by Anderson et al. [14], which provide one of the few national samples of gig workers in the United States. Specifically, we use data from Wave 94 of their American Trends Panel that was conducted from August 23−29 in 2021. This research was approved by the Purdue University IRB #: IRB-2024-1253.

To complete wave 94, Pew contacted 11,178 members of the American Trends Panel (ATP), who are recruited through national, random sampling of residential addresses [14]. Ultimately, 10,348 respondents completed the survey for a response rate of 93%. Respondents were asked separate questions to determine if they had ever done five different types of gig work (ride-hailing, shopping & delivering groceries, household tasks, food-delivery, and other). They were also asked if they had ever done gig work that involved delivering packages using a personal vehicle. Following analyses by researchers at Pew [14], we counted respondents who had done any of those six things as having done gig work. Our initial sample thus included 1,301 respondents who had ever done gig work.

For this analysis, we also drop respondents who reported their gender as “Other” and respondents who listed their race as Asian or “Other.” These categories are either ambiguous or too small to support an intersectional analysis of race, age, and gender. Together, these restrictions reduced our sample size by 111. We lost an additional 110 respondents who were missing on one or more variables in our analysis. Our final sample size was 1,080.

### Measures

#### Harassment.

In the ATP survey, respondents who indicated that they had done gig work at some point were asked, “Since you first started taking on these kinds of jobs, how often, if ever, have you experienced each of the following while completing jobs via these apps or websites?” The items were, “Felt unsafe,” “Been treated rudely,” and “Experienced an unwanted sexual advance.” Together, the three items capture a wide range of harassment behaviors. For each item, respondents were coded 1 if they indicated they had “Never” experienced that form of harassment, 2 for “Rarely,” 3 for “Sometimes,” and 4 if they reported “Often” experiencing harassment. We took the average of the three items to make an overall measure with higher values indicating more harassment. The Chronbach’s alpha for the index is 0.78. (Respondents who had not done gig work were not asked these questions.)

#### Race, gender, and age.

Researchers at Pew combined information from a question about Hispanic identity and a question about race to create a variable that classifies respondents as white non-Hispanic, Black non-Hispanic, Hispanic, Asian non-Hispanic, or Other. We use this variable to measure race, but as mentioned above, we dropped respondents in the last two categories, because they were not large enough to support an intersectional analysis incorporating race, gender, and age. We use White respondents as the reference category. To indicate gender, respondents choose from three options: man, woman, and other. We dropped the third category that was too small to analyze, leaving man and woman, with man as the reference category. Finally, respondents reported their age by selecting one of four options: 18–29, 30–49, 50–64, or 65 + . We use 18–29 as the reference category.

#### Controls.

We also control for several factors that might be related to both harassment and our focal variables. Respondents indicated if they were born in the U.S., Puerto Rico, another U.S. territory, or in another country. We re-classified respondents who were born in Puerto Rico or another U.S. territory as born in the U.S. to produce two categories: born in the U.S. (coded 1) and born in another country (coded zero). Respondents also reported if they were U.S. citizens (coded 1) or not U.S. citizens (coded zero) and their preference for Spanish (coded 1) or English (coded zero). We included these controls because previous research indicates that in the conventional workplaces, immigrants tended to experience higher levels of workplace discrimination than native workers [67]. We also included a control for family income because past research indicates that low-income respondents experienced high levels of harassment [68]. Respondents were grouped into nine income categories: less than $30,000, $30,000-$39,999, $40,000-$49,999, $50,000-$59,999, $60,000-$69,999, $70,000-$79,999, $80,000-$89,999, $90,000-$99,999, or $100,000 or more. We use less than $30,000 as the reference category. We also identify respondents who only did gig work online (as opposed to in-person). We identified these respondents based on their reports of the different types of gig work they had done. Specifically, respondents who did a type of gig work that the survey did not mention explicitly were allowed to write in the type of gig work they did. We were able to classify these “other” types of gig work into four categories: online tasks, household repair work, caregiving, and online selling. We use all this information together with the reports of other types of gig work to identify respondents who had only done online gig work. We added this control based on the idea that respondents who only did online gig-work might be less likely to experience harassment than people who interacted with customers face-to-face. We also control for the region of the U.S. where respondents lived (Midwest, South, or West, with Northeast as the reference category). Finally, we control for whether respondents were living with a partner and if they were college graduates.

### Analytic plan

To determine how harassment varies by gender, race, and age when examined independently, we conduct t-tests of means on the harassment index (Table 2). This helps test Hypothesis 1. To test Hypotheses 2, 3, and 4, which are motivated by the intersectional perspective, we use a set of figures and nested OLS regressions. Fig 1 shows the mean level of harassment for each race-gender group, thus providing a partial assessment of (H2). Fig 2 shows the mean level of harassment for each gender-age group, thus providing a partial assessment of (H3). Fig 3 shows the mean level of harassment for each race-age group, thus providing a partial assessment of (H4). We use nested OLS regressions with interactions and controls to assess the robustness of the patterns from the figures. Figs 4 and 5 plot predictions from our final regression model to clarify the meaning of the interactions. All results are adjusted using weights supplied with the data.

**Fig 1:**
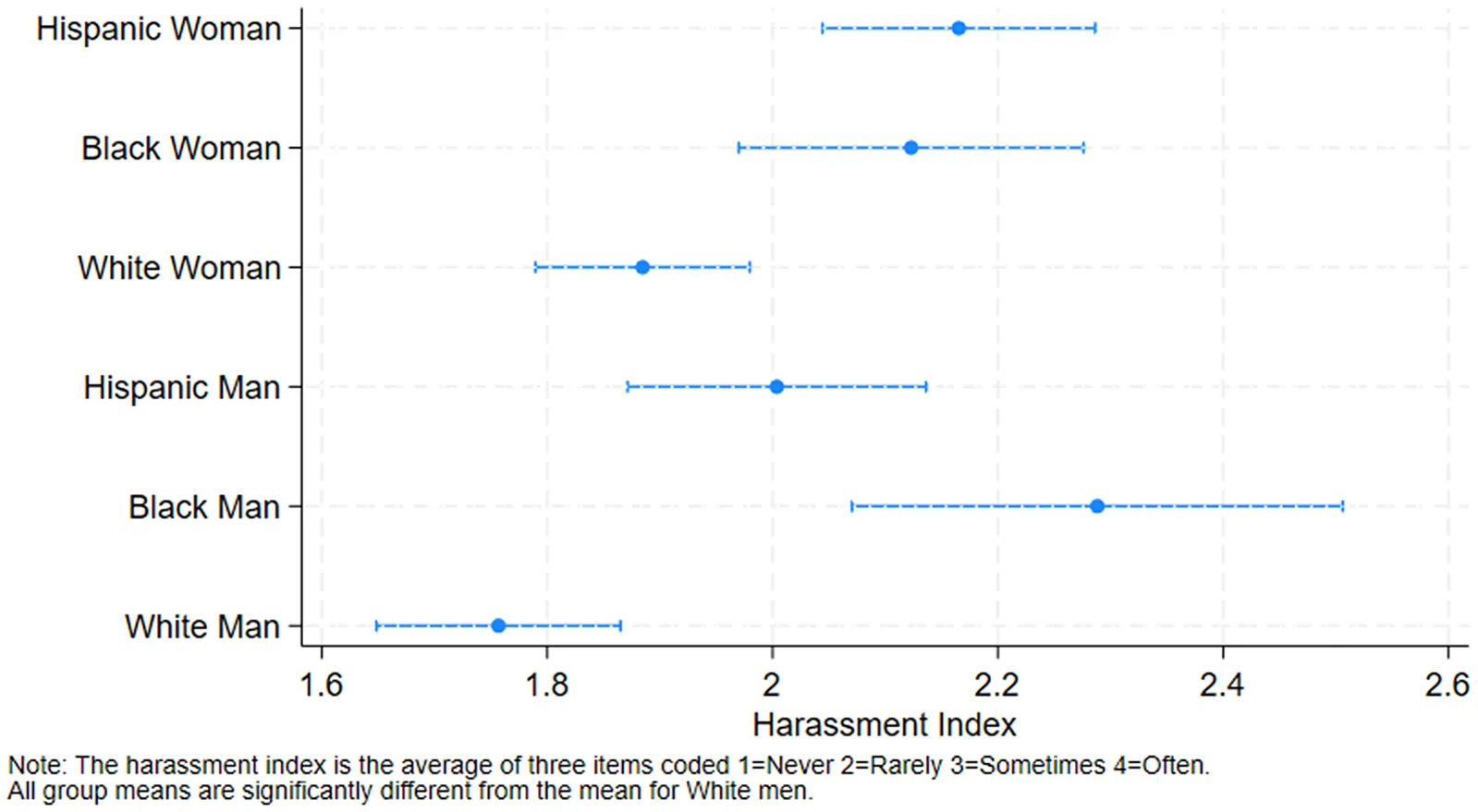
Gig workers’ experiences of harassment by race-gender.

**Fig 2:**
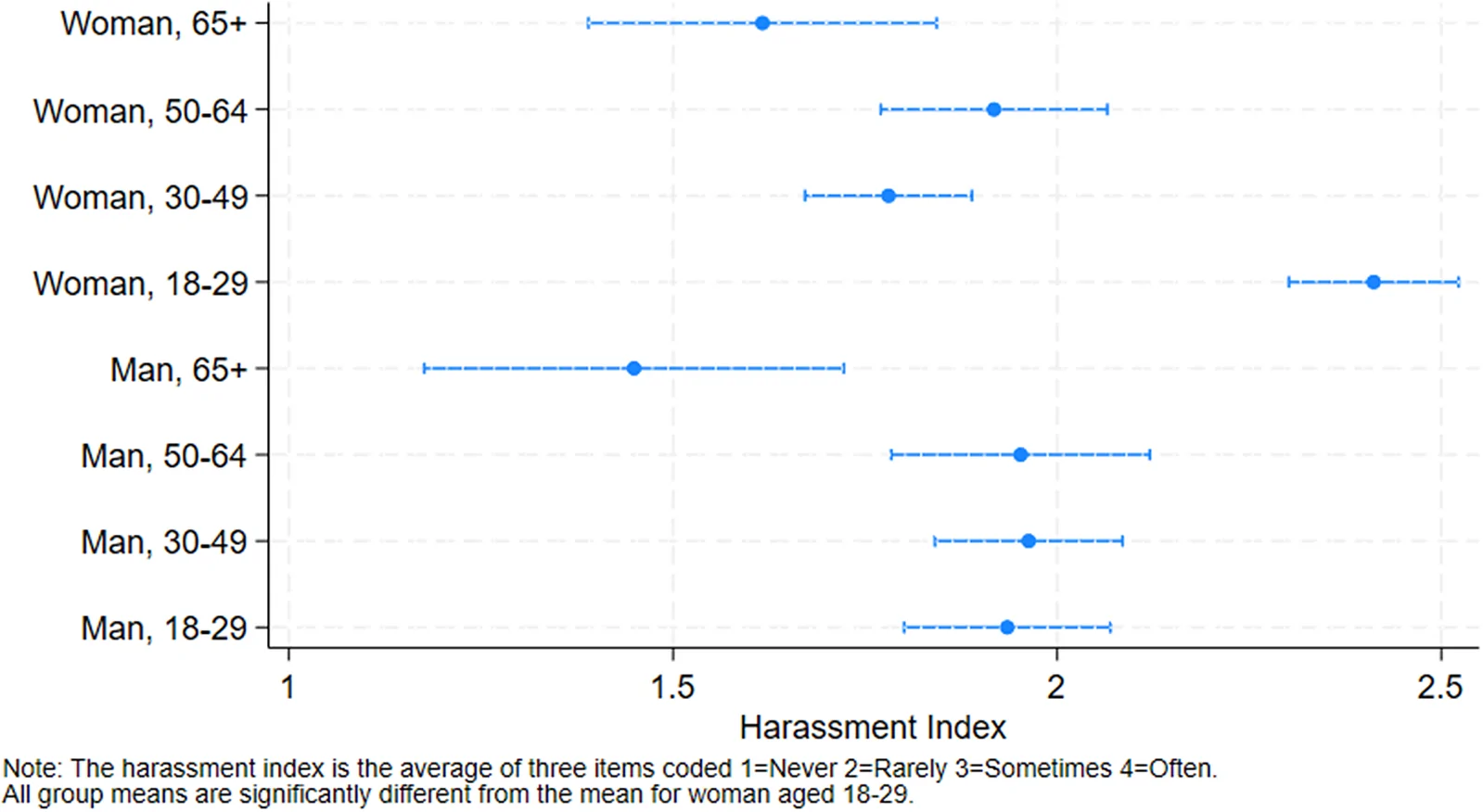
Gig workers’ experiences of harassment by gender-age.

**Fig 3:**
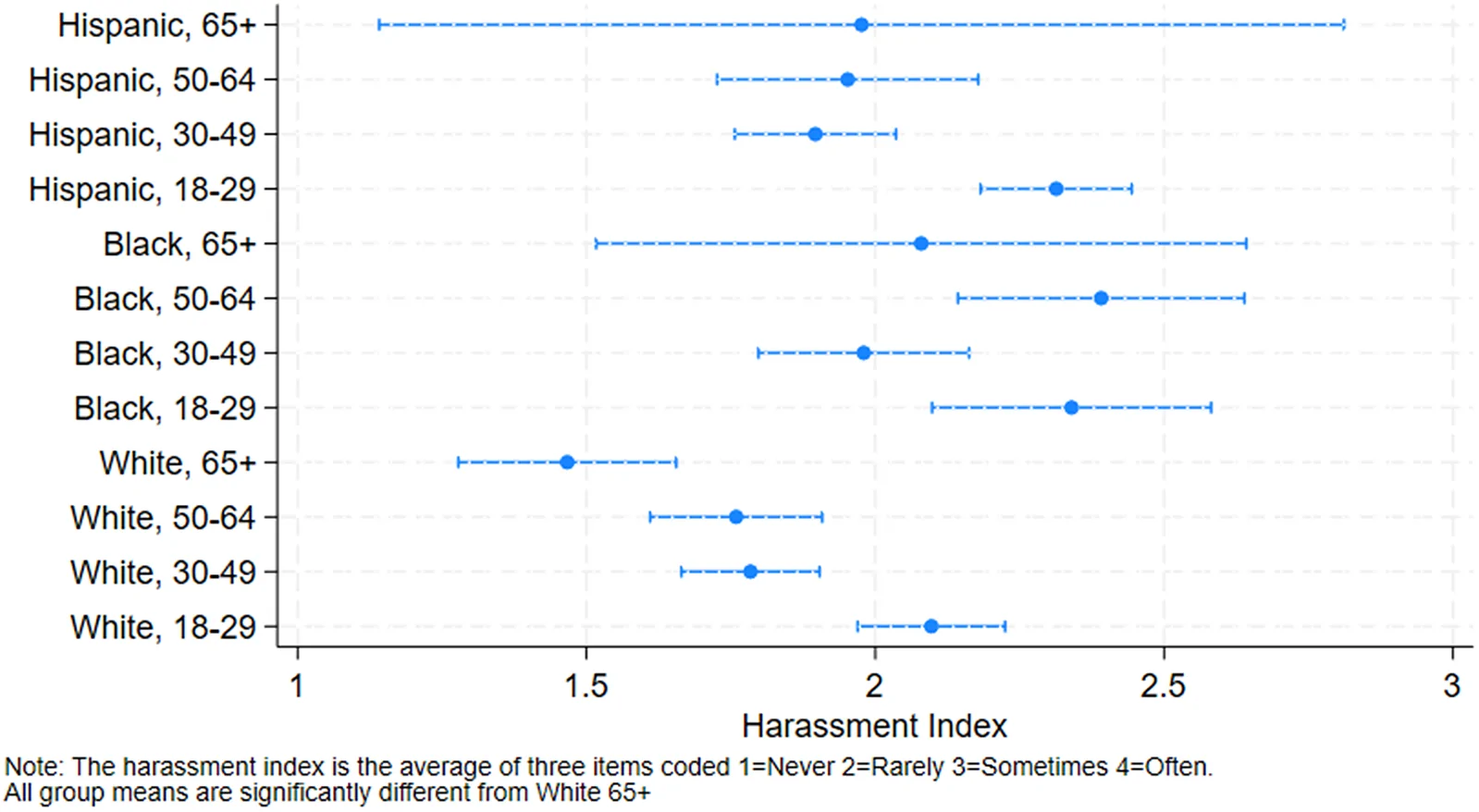
Gig workers’ experiences of harassment by race-age.

**Fig 4:**
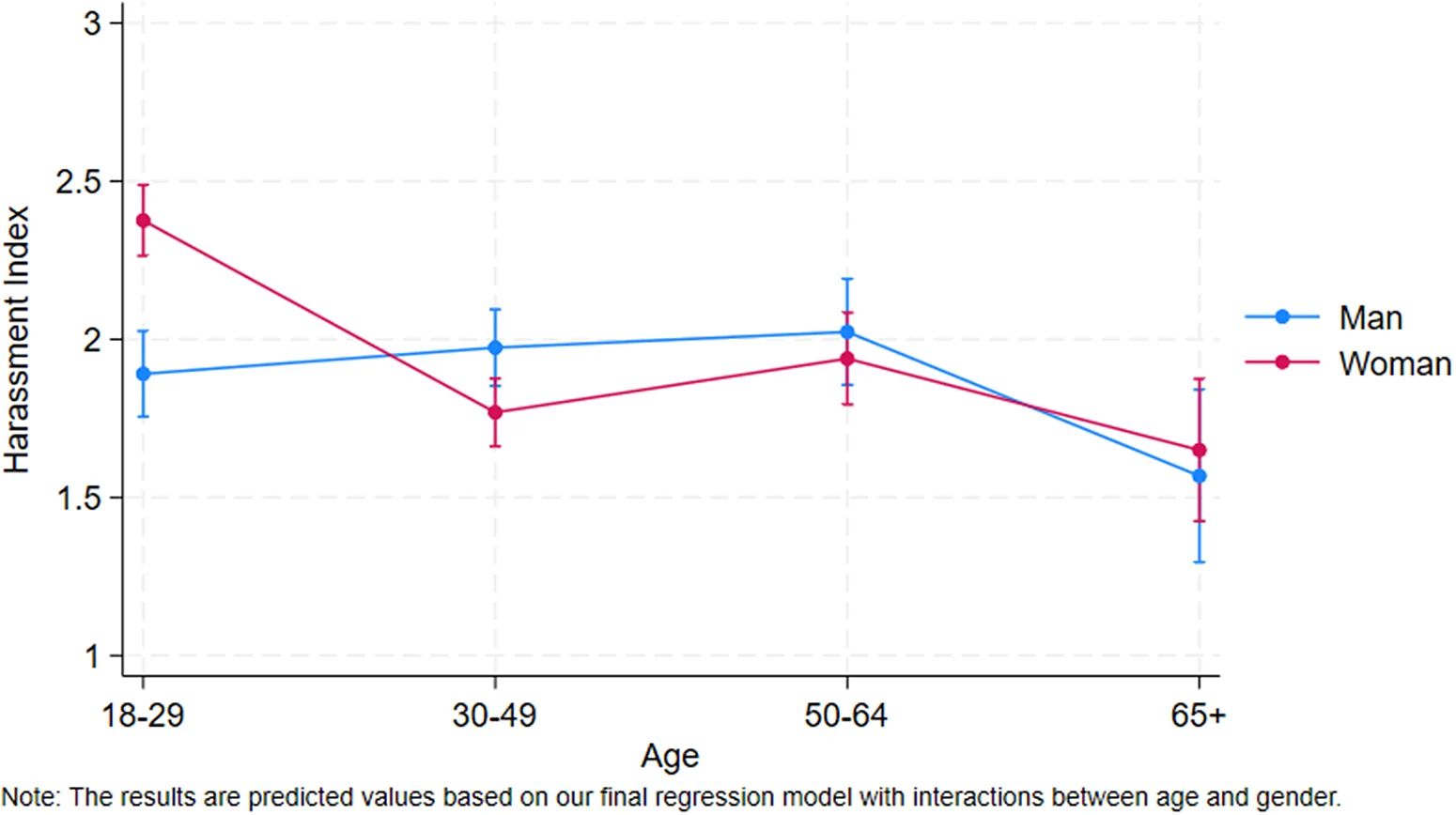
Predicted harassment by gender-age.

**Fig 5:**
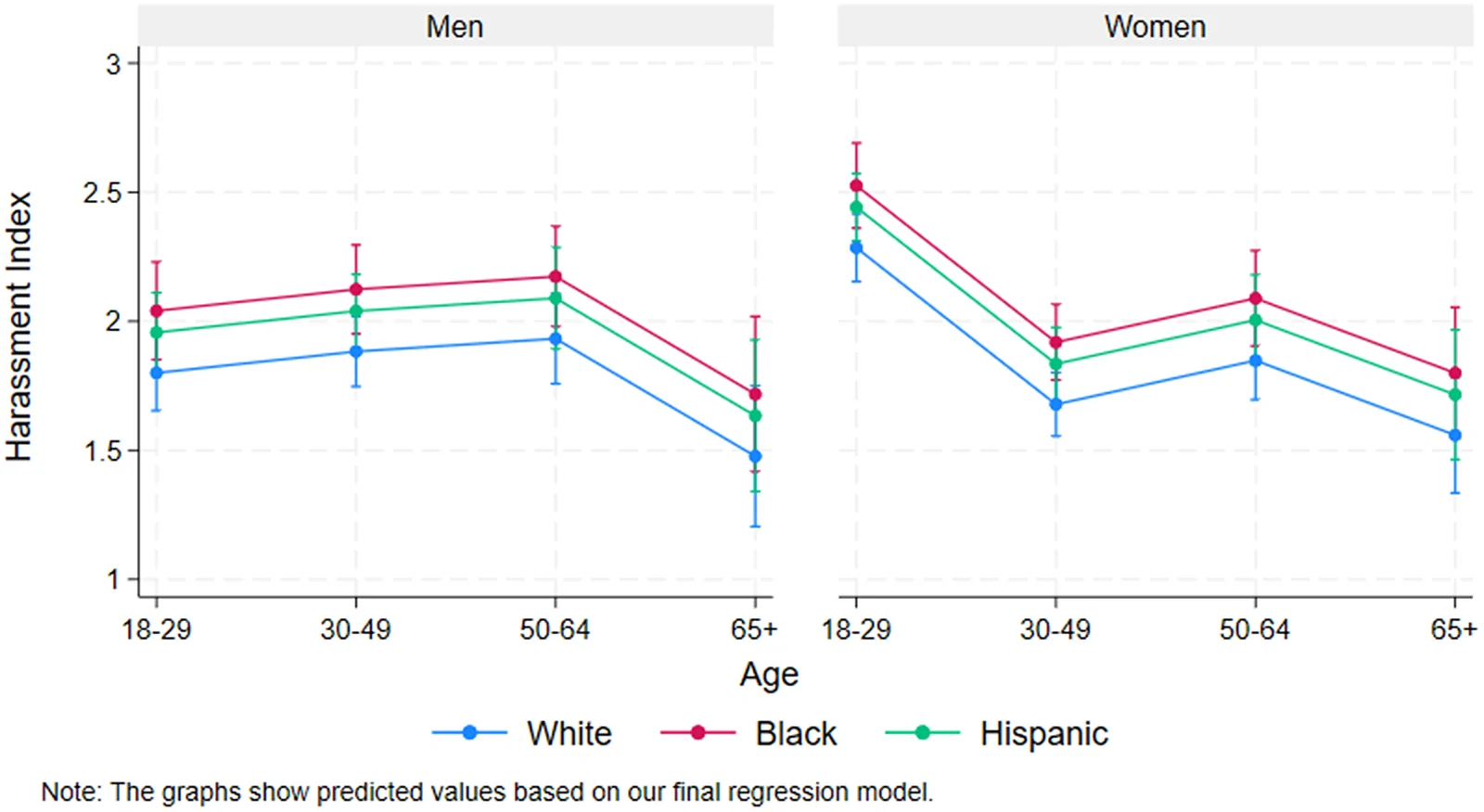
Predicted harassment by race-age for men and women.

### Analysis

Table 1 shows the basic demographics of our sample before accounting for intersecting identities. The sample is 57.6% women and 50.9% White. The largest age group is the 30–49 year-olds, who are just over 37% of the sample. We also calculated the percentage of respondents in a more intersectional way to identify race-gender, race-age, and gender-age groups. Of the six race-gender categories, the largest was White women (28.7%) and the smallest was black men (5.5%). Cross-classifying race and age resulted in twelve groups of respondents with White respondents ages 30–49 being the largest category (17.4%) and Hispanic respondents ages 65 + the smallest (0.4%). Finally, in the eight possible gender-age categories, women ages 30–49 were the largest group (21.0%) and men ages 65 + were the smallest (3.3%). Given the small number of Black and Hispanic respondents in the 65 + category, we cannot produce very precise estimates of harassment for those groups, but all other combinations contain larger numbers of respondents. Table 1 in S1 Appendix shows weighted and unweighted percentages and N for every cross-classification of race, gender and age.

**Table 1:** Sample demographics.

	Percent	N
Gender		
Man	42.4	458
Woman	57.6	622
Race-Ethnicity		
White	50.9	550
Black	16.6	179
Hispanic	32.5	351
Age		
18-29	34.1	368
30-49	37.6	406
50-64	20.1	218
65+	8.1	88
Total		1,080

The mean score on the harassment index is 1.97, but there are differences across groups. Our t-tests indicate that women report significantly more harassment than men: 2.02 vs 1.91 (Table 2). Black and Hispanic respondents report statistically higher levels of harassment than White respondents. Finally, respondents aged 18–29 report more harassment than older respondents. Together, these patterns for gender, race, and age provide substantial support for Hypothesis 1. A closer look at the size of the differences in each section of Table 2 suggests that although there are differences in harassment by gender, the differences by race and age are more pronounced.

**Table 2:** Mean level of harassment by gender, race, and age.

	Harassment Index
Total	1.97
Man (Reference category)	1.91
Woman	2.02*
White (Reference category)	1.83
Black	2.18***
Hispanic	2.09***
18-29 (Reference category)	2.22
30-49	1.86***
50-64	1.93***
65+	1.55***

The harassment index is the mean of three variables coded 1 = Never 2 = Rarely 3 = Sometimes 4 = Often.

Stars indicate statistically significant differences from the reference group based on a t-test.

***=p<0.001 **=p<0.01 *=p<0.05.

Table 2 does not report the percentage of each group reporting any harassment. Such percentages are easy to grasp. For instance, while 70% of White gig workers report being harassed, 82% of Black and 79% of Hispanic gig workers report being harassed. Such percentages, however, are likely to underestimate group differences by discarding information about the frequency of harassment. We thus focus instead on mean differences on the harassment index (See S1 Appendix for comments about this issue and a few more percentages.)

To examine harassment in a more intersectional way, we graph mean levels of harassment for each two-way cross-classification of race, gender and age, i.e., by race-gender, gender-age, and race-age. Fig 1 shows how harassment differs across race-gender categories. Contrary to Hypothesis 2, Black and Hispanic Women do not report more harassment than all other groups. Rather, Black women and Hispanic women report significantly more harassment than White men and White women but similar levels of harassment as Black men and Hispanic men. In other words, we do not find a double disadvantage for Black or Hispanic women. Still, this is primarily because Black men report comparatively high levels of harassment. Indeed, although Black men are not typically described as dually disadvantaged, they report levels of harassment that are similar to (in fact slightly higher than) those of Black women. Hispanic men, in contrast, report the least harassment of any non-white group. These contrasting patterns for Black and Hispanic men suggest that perhaps the relationship between gender and harassment varies by race. The gender differences, however, are not large for any racial group. Furthermore, these patterns should be interpreted cautiously because they may change when accounting for race, gender, and age simultaneously or after controlling for other factors. Moreover, the group that really stands out in Fig 1 is White men. Being both White and a man is associated with distinctly low levels of harassment.

Fig 2 shows harassment across gender-age groups, and it indicates that women ages 18–29 report significantly more harassment than any other gender-age group. The oldest men and women report the least harassment. This provides strong support for Hypothesis 3. Being a young woman is associated with distinctly high levels of harassment. The figure also suggests that the connection between gender and harassment may vary by age such that it is strongest among young respondents. We re-visit that topic in our regressions that account for race, gender, and age simultaneously.

Fig 3 provides the final two-way cross-classification by examining harassment in race-age categories. One notable pattern is that White respondents aged 65-years or older report significantly less harassment than all other race-age groups. The results for the oldest Black and Hispanic respondents have large confidence intervals that reflect the small sample sizes for those groups, but the differences between them and the oldest White respondents are still significant. Hypothesis 4 predicted that young Black and Hispanic workers would report more harassment than other race-age groups. That prediction is not supported. Those two groups report high levels of harassment, but they do not stand out from all other groups. Rather, Fig 3 underscores a connection between age and harassment, particularly among White and Hispanic respondents, where the youngest respondents report more harassment than older respondents of the same race. The primary exception to this age-related pattern appears among Black respondents, where levels of harassment are fairly high for all age groups. This hints that being Black may be strongly connected to harassment in a way that mutes age differences. Once again, these results should be interpreted with caution. Our regressions may reveal other patterns. Overall, however, Fig 3 provides little support for Hypothesis 4. The youngest Black and Hispanic workers do not report significantly more harassment than other race-age groups.

Our regressions re-examine how harassment is connected to race, gender, and age in a multivariate context. We begin with simple models that do not account for potential interactions among race, gender, and age. In Model 1 of Table 3, we predict harassment using only race. On average, both Black and Hispanic respondents report more harassment than White respondents. In Model 2, we add gender and find that, on average, women do not report significantly more harassment than men after accounting for race (the results for race, however, remain significant). In Model 3, we add age variables. The youngest respondents report more harassment than older respondents.

**Table 3:** OLS regressions predicting harassment levels.

	Model 1	Model 2	Model 3	Model 4	Model 5	Model 6	Model 7
Black	0.349***	0.339***	0.333***	0.251***	0.408**	0.199	0.241**
Hispanic	0.263 ***	0.265***	0.189**	0.188**	0.158	0.170	0.157*
Woman		0.095	0.083	0.069	0.082	0.079	0.485***
30-49			−0.363***	−0.315***	−0.309***	−0.285**	0.083
50-64			−0.275***	−0.200**	−0.208**	−0.280**	0.133
65+			−0.589***	−0.559***	−0.557***	−0.621***	−0.323*
Control Variables							
Prefers Spanish				−0.285*	−0.278*	−0.292*	−0.340**
College Graduate				−0.102	−0.095	−0.112	−0.113
Born in US				−0.095	−0.081	−0.096	−0.160
US Citizen				0.051	0.052	0.053	0.059
Living with Partner				−0.004	0.001	−0.003	−0.030
$30,000-$39,999				−0.060	−0.060	−0.047	−0.057
$40,000-$49,999				−0.094	−0.094	−0.086	−0.058
$50,000-$59,999				−0.205*	−0.194*	−0.195*	−0.199*
$60,000-$69,999				−0.002	0.004	0.011	−0.003
$70,000-$79,999				−0.410**	−0.408**	−0.392**	−0.347**
$80,000-$89,999				−0.523***	−0.515***	−0.531***	−0.556***
$90,000-$99,999				0.041	0.042	0.033	0.090
$100,000 or more				−0.228*	−0.228*	−0.212*	−0.230*
Midwest				0.137	0.146	0.144	0.100
South				0.139	0.149	0.142	0.153*
West				−0.030	−0.031	−0.024	−0.033
Only did online work				−0.437*	−0.441**	−0.420*	−0.381*
Interactions							
Black # Woman					−0.234		
Hispanic # Woman					0.068		
Black # 30–49						−0.107	
Black # 50–64						0.316	
Black # 65+						0.345	
Hispanic # 30–49						−0.020	
Hispanic # 50–64						0.068	
Hispanic # 65+						0.432	
Woman # 30–49							−0.690***
Woman # 50–64							−0.570***
Woman # 65+							−0.403*
Constant	1.829***	1.776***	2.048***	2.146***	2.111***	2.145***	1.971***
N	1080	1080	1080	1080	1080	1080	1080
R^2^	0.029	0.032	0.078	0.128	0.131	0.134	0.156

***=p<0.001 **=p<0.01 *=p<0.05.

These patterns are robust to the inclusion of control variables. In Model 4, differences in harassment by race are still significant. This indicates that racial differences in the earlier models were not driven by any of the control variables. The three age coefficients also remain significant. Overall, these models partially echo Table 2 and provide some support for Hypothesis 1: non-white and young respondents experience high levels of harassment even after the inclusion of control variables. Gender differences, in contrast, are not significant. This pattern departs somewhat from Table 2, but it echoes the generally weak findings regarding gender from several meta-analyses [17,24,33,34].

The remaining regressions include interactions among our focal variables, and they suggest that in the context of gig work, both gender and age are related to harassment in similar ways across racial groups. The interactions in Model 5 examine if gender differences in harassment vary by race. The non-significant interactions indicate that gender differences in harassment among Black and Hispanic respondents resemble those among White respondents (i.e., race does not moderate the connection between gender and harassment). The interactions in Model 6 examine if age differences in harassment vary by race. Those interactions are not significant either, indicating that the connection between age and harassment is similar across racial groups. Overall, these regressions thus fail to support Hypothesis 2 that predicted a “double jeopardy” for Black women and Hispanic women. The regressions also fail to support Hypothesis 4, which predicted especially high harassment among young Black and young Hispanic gig workers.

To make sure these non-significant interactions in Models 5 and 6 are not an artifact of our model specification, we estimated some supplemental regressions. We collapsed the top four income categories and removed the controls for region to see if the interactions in Models 5 and 6 would be significant in simpler models. They were not. We also tried adding both sets of interactions separately to Model 3, which does not contain any control variables. F-tests indicated that the race-gender and race-age interactions did not significantly improve the model. Together, these results indicate that although Figs 1 and 3 hinted at potential moderating effects of race, those patterns are not significant when race, gender, and age are examined together (even without controls). Rather, our analysis indicates that gender and age are related to harassment in similar ways for White, Black, and Hispanic respondents. This does not mean that race is not an important predictor of harassment. On the contrary, we find that the connection between race and harassment is present regardless of age or gender.

Our regressions provide strong evidence that harassment reflects the intersection of gender and age. In Model 7, all three interactions between gender and age are significant, indicating that the connection between gender and harassment varies by age. The coefficient for woman (0.485) takes on a new meaning with the inclusion of the interactions. It indicates that women ages 18–29 report significantly more harassment than men of that age. The interactions indicate that the gender difference in harassment is significantly weaker for people in the 30–49, 50–64, and 65 + age groups than for those aged 18–29. With the inclusion of the gender-age interactions, the main effects for age show the patterns of harassment by age among men. They indicate that levels of harassment do not vary much with age among men. The exception is men aged 65 + , who report significantly less harassment than younger men. Overall, these results strongly support Hypothesis 3. Supplemental analyses indicate that the patterns of significance are similar if we remove sexual harassment from the harassment index or examine the three items in the index as separate dependent variables (Tables available upon request).

Fig 4 helps clarify the results from Model 7 by plotting predicted harassment by age and gender while adjusting for all the other covariates. The most striking pattern is that young women aged 18–29 experience more harassment than all other age-gender groups. Furthermore, the connection between gender and harassment is not uniform. Gender differences in harassment are small in the older age groups. Men and women in the 50–64 and 65 + category, for instance, report nearly identical levels of harassment. It is only among the two youngest age groups (30–49 and 18–29) that gender differences are significant, and the difference is especially large for the youngest age group. Finally, although it is useful to examine the significant differences in Fig 4, it is also instructive to note similarities. In particular, among men, harassment does not vary much by age. The only men who report significantly different levels of harassment than other men are men who are 65 + . In short, Fig 4 reveals an intersectional pattern: it indicates that harassment cannot be understood well by examining gender and age separately. Gender differences in harassment are often small, and variations by age are often modest, but young women stand out due to their high levels of harassment. These results support Hypothesis 3: gig workers who are both women and young experience more harassment than others.

Fig 5 provides a comprehensive summary of harassment by gender, age, and race. The non-significant interactions with race in our regressions indicate that Black and Hispanic respondents reported significantly more harassment than White respondents regardless of their gender or age. Consequently, the predictions in Fig 5 (based on Table 3 Model 7) show a uniform pattern in which Black and Hispanic gig workers report more harassment than their White counterparts across all gender-age groups, while also reflecting the intersectional relationship between gender and age. The net effect is that harassment is lowest for the oldest White men and women (see the blue lines at 65+), and it is highest for women under 30, especially if they are Black or Hispanic (see the lines for women ages 18–29).

Finally, it is important to note that the connection between gender-age groups and harassment is much stronger than other statistically significant relationships. The coefficient for woman (0.485 from Table 3 Model 7) describes the gender difference in harassment between men and women ages 18−29. The equivalent standardized coefficient (0.277) is over four times larger than the standardized coefficient for doing only online gig work (−0.381 standardized is −0.066). This suggests that for gig workers under 30, being a woman is associated with an increase in harassment that is more than quadruple the difference between people who only do on-line gig work and those who do in-person gig work. Partial omega squared statistics provide another way of assessing effect sizes. Doing only online gig work is associated with a statistically significant reduction in harassment (which is notable because there are only 36 workers of this type in our analysis), but it only explains about 0.39% of the variance in harassment. Race explains 0.9%, and income about 1.66%. Gender-age groups, in contrast, explain 5.77% of the variance in harassment.

## Discussion

Although many people are attracted to gig work by the opportunity to be their “own boss” and set their own schedules, gig work also carries a number of risks [3,4]. Scholars have emphasized the lack of benefits and minimum wage guarantees as well as the lack of protections against discrimination and harassment [1]. We offer the first in-depth quantitative examination of harassment among gig workers across the U.S., and we extend previous work by using an intersectional lens.

Without organizational or legal protections, gig workers risk harassment by customers and the public, including rude treatment, unwanted sexual advances, and threats. Qualitative research has explained why gig workers are so vulnerable and how they try to protect themselves from harassment [10,12,13]. A quantitative report by the Pew Research Center examined specific types of harassment and variations by race, gender, and age [14]. The patterns provided some support for predictions from the perpetrator-predation paradigm: people of color, women, and young workers report especially high levels of harassment.

We build on existing studies of gig workers by examining how harassment (measured broadly) varies across the intersection of race, gender, and age. Recent meta-analyses and reviews found mixed evidence of gender and race differences in harassment among workers in conventional jobs [17,24,33,34]. However, we thought the lack of protections against harassment might produce clearer demographic differences in harassment among gig workers by creating a context that emboldens perpetrators, who are inclined to harass people who belong to groups that are perceived to be vulnerable. Furthermore, because existing research on harassment among conventional and gig workers typically examines race, gender, and age separately, we suspected that it might have overlooked important but more complex patterns. Intersectional theory suggests that people with multiple disadvantaged identities (e.g., Black and woman, young woman, etc.) often experience worse outcomes than others due to a “double jeopardy” [25,65]. It also suggests that race, gender, and age may produce distinct outcomes for other groups like Black men or Hispanic men.

Using survey data from wave 94 of the American Trends Panel, we analyzed harassment in gig work and focused on three intersections: race-gender, age-gender, and age-race. We predicted that people of color, women, and young workers would experience high levels of harassment. We also predicted that Black women and Hispanic women, young women, and young Black or Hispanic workers would experience especially high levels of harassment due to their dually disadvantaged identities. Examining these patterns is especially important because participation in the gig economy is high among people of color, women, and young people. This could concentrate the risks of gig work among those groups.

Our results provide strong evidence of particular demographic differences in harassment and also evidence of intersectionality. In our initial bivariate analysis, women, young workers, and people of color reported more harassment than their counterparts (men, older workers, and White gig workers). Our comparisons of mean harassment at the two-way intersections of race-gender, gender-age, and race-age also hinted at some potential interactions of race with gender or age. Our regressions, however, which examined race, gender, and age simultaneously and included control variables to help isolate the effects of our focal variables, reveal a different picture. Black and Hispanic workers reported more harassment than White workers, and young workers reported more harassment than older workers, but women did not consistently report more harassment than men. These patterns echo the muted gender differences in harassment reported in some meta-analyses. However, we also found that gender differences in harassment were particularly large among the youngest gig workers even though gender differences were rather small for older workers. Furthermore, young women reported significantly more harassment than any other age-gender group, even after controlling for other factors. In short, we found that the connection between gender and harassment varies by age. That might help explain why previous studies that examine the average effect of gender found much smaller differences.

Contrary to our expectations, gender and age variations in harassment did not differ much by race. Still, Black and Hispanic workers reported more harassment than White workers whether they were men or women, young, or old. Thus, like Berdahl and Moore [65], we find that race has an additive rather than a multiplicative effect. Young Black and Hispanic women do face distinctly high levels of harassment due to their location on the axes of race, gender, and age, but we did not find that gender or age had a different connection to harassment for any particular racial group. Our two central findings are thus that young women of all races stand out for their frequent experiences with harassment, and that harassment is higher among Black and Hispanic workers than among White workers. Together, this means that young women of color experience the most harassment.

We cannot test directly if harassment is more common in gig work than in conventional work because respondents were only asked about harassment in gig work. The perpetrator-predation framework suggests that gig workers might experience more harassment than conventional workers on average. Without workplace or legal protections, co-workers who might defend them, incentives to “brush off” harassment, and isolated work locations, they may be easier and thus more frequent targets for perpetrators [10,12,13]. Workers in some conventional jobs (e.g., food industry), however, also face high levels of harassment [69], as do low wage workers in general [19]. The Pew data do not allow for comparisons with those groups (See S1 Appendix for additional comments about this issue). Still, we did find an intriguing difference among gig workers: People who only did online gig work reported less harassment than other gig workers. Future studies should examine how harassment varies across different types of gig work in more detail (especially online vs. location-based gig work). Existing research suggests that workers in different types of gig work (e.g., drivers, domestic workers, and online freelancers) may face unique and potentially different risks of harassment [10,12,13,53–55,70]. Future studies should also gather data that allow direct comparisons of harassment in gig work and conventional jobs as a whole and within occupations that exist in both the conventional and the gig economy (e.g., taxi drivers, delivery drivers).

### Practical implications

Despite this limitation, our analysis highlights patterns that are important for social and organizational policy. Policy makers and gig platforms should consider the harassment that gig workers experience (especially those who are young White, Black, or Hispanic women) and draw on those patterns and workers’ insights to design solutions. Currently, many gig workers lack both methods for reporting harassment and the legal protections that conventional workers have before and after reporting harassment. This is because gig workers are typically classified as independent contractors rather than employees [1]. Some platforms have tried to improve the situation. Uber, for instance, provides drivers with an emergency assistance button; a way to report incidents 24/7; the ability to: share their location with friends and family, make audio recordings, and rate passengers; and the RideCheck tool, which monitors if a ride is going according to plan [71]. In 2026, Uber also introduced a feature that allows women drivers to request women passengers and vice versa [72]. Still, these features rely heavily on women to do the work of protecting themselves, and many only address harassment after it happens [10]. This reflects the general U.S. legal approach to harassment, which focuses on responding to harassment rather than on proactive measures like those that the European Union and the United Kingdom use to prevent harassment [73,74]. Qualitative studies of the United States reveal that many gig workers find the steps taken by platforms inadequate [10,12,13]. Our analysis corroborates this finding by showing that harassment remains a problem across the gig economy. Scholars recommend additional changes such as allowing more workers to rate customers, better dispute resolution options, clearer rules regarding performance requirements [54,75], and worker-inspired solutions, particularly those that are proactive and intended to reduce inequality (e.g., better ways to avoid, flag, or ban abusive customers) [76]. Our results suggest that policy makers and organizations should consider these options and pay special attention to the experiences of young women.

### Theoretical contributions

Our analysis also corroborates several theoretical arguments about harassment. Our results support the perpetrator-predation paradigm, which suggests that lower-status people (e.g., young women and people of color) are especially likely to be harassed [24,30,33]. In light of the mixed evidence of gender, race, and age differences in harassment in studies of conventional workers, the relatively strong patterns we found highlight a potential connection between precarious work and demographic differences in harassment [7,19–21]. Our results also support the theoretical claim that patterns of harassment reflect workplace context [17,24,33].

Furthermore, our results underscore the importance of examining harassment using an intersectional perspective [7,16–18]. We found that among gig workers in the U.S., the connection between gender and harassment varies substantially by age such that young women report particularly high levels of harassment. If we had examined gender differences without considering how gender intersects with age, we would have erroneously concluded that gender is not related to harassment. Our regression analysis did not suggest that patterns of harassment by gender or age are different for different racial groups. Gender differences in harassment, for instance, were similar for White, Black, and Hispanic respondents. However, examining harassment at the intersection of race, gender, and age still led to important insights. Specifically, we found that young Black and young Hispanic women report the most harassment because of their location at the intersection of gender-age combined with the robust effect of race. If we had examined race, gender, and age separately (rather than as identities that people hold simultaneously), we would have overlooked that pattern. In short, our results suggest that taking an intersectional perspective can help uncover complex patterns of harassment among gig workers. This is the point of the intersectional perspective: it focuses on guiding the questions researchers ask rather than on hypothesizing what they will find [77]. Consequently, our conclusion that race does not moderate the effects of gender or age does not call the intersectional perspective into question. The theoretical framework does not hypothesize a universal “double jeopardy” for Black women or particular outcomes for other race-gender groups. Rather it argues that research should be done in ways that can uncover such patterns when they exist. In this spirit, we encourage additional intersectional analyses of harassment among workers in other non-standard work arrangements.

### Prospects for the future

By shedding new light on the harassment of gig workers, our analysis underscores a continued need for reform. Previous studies show that many gig workers face risks because of low and unstable pay and their lack of access to fringe benefits [1,45,47]. Our analysis corroborates qualitative research suggesting that they also face a notable risk of harassment. The best way to address this risk may not be to oppose gig employment. That could have a negative financial effect on many people who rely on it for supplemental income [43,47,76]. Instead, perhaps policymakers should focus on improving gig platforms and labor laws to reflect the concerns raised by gig workers [78,79]. It would also be helpful to improve the quality of conventional jobs so fewer people need gig employment to make ends meet [47].

Our sense is that even if some law makers or employers in the United States favor the status quo, the sheer number of sexual harassment complaints in the gig economy and the associated court cases may eventually lead to change. Whether such changes will provide substantial protection for gig workers is unclear. On average, from 2017 to 2022, Uber received a report of sexual misconduct or assault in the U.S. every eight minutes, and it faces thousands of lawsuits and pressure from voters and lawmakers [80]. Many times, drivers are the perpetrators, and although Uber has long claimed in court that it is not responsible for the actions of its drivers, others disagree. In February of 2026, a jury in Arizona held Uber responsible for the behavior of a driver who raped a passenger, potentially opening the door for liability of gig platforms in future cases [81]. Battles over legal protections for drivers and other gig workers, in contrast, continue to favor gig platforms. Legislation introduced in 2018 that would extend federal employment laws to cover independent contractors has still not passed [75]. In fact, in early 2026, the U.S. Department of Labor proposed a rule that would classify even more workers as independent contractors [82]. Such developments suggest that although gig platforms may soon need to provide more protection for customers, substantial improvements for gig workers may still be years away. Until then, gig work may remain both economically precarious and associated with a heightened risk of harassment for young women, especially young women of color.

## Supporting information

S1 Appendix S1 Table 1. Demographic Cross-classifications. S1 Table 2. Proportion Harassed by Gender, Race, and Age. (DOCX)
