# Supplementary material for: Young women at risk: Harassment by gender, race, and age in the gig economy
Source: PLoS One. 2026 Nov 4;21(11):e0359956. doi: 10.1371/journal.pone.0359956 (PMC13734466; doi:10.1371/journal.pone.0359956)
Supplement: Supplementary file 1 [file pone.0359956.s001.docx]

**S1 Appendix**

| S1 Table 1. Demographic Cross-classifications | | | | |
| --- | --- | --- | --- | --- |
|  | Weighted | | Un-weighted | |
|  | Percent | N | Percent | N |
| Race and Gender |  |  |  |  |
| White Man | 22.2 | 239 | 23.7 | 256 |
| Black Man | 5.5 | 59 | 3.1 | 34 |
| Hispanic Man | 14.8 | 160 | 13.0 | 140 |
| White Woman | 28.7 | 310 | 31.8 | 343 |
| Black Woman | 11.1 | 120 | 10.3 | 111 |
| Hispanic Women | 17.7 | 192 | 18.1 | 196 |
| Race and Age |  |  |  |  |
| White 18-29 | 15.3 | 165 | 9.0 | 97 |
| White 30-49 | 17.4 | 188 | 20.2 | 218 |
| White 50-64 | 11.2 | 121 | 15.6 | 169 |
| White 65+ | 7.0 | 76 | 10.6 | 115 |
| Black 18-29 | 4.3 | 46 | 1.8 | 19 |
| Black 30-49 | 7.5 | 81 | 6.9 | 74 |
| Black 50-64 | 4.0 | 44 | 3.7 | 40 |
| Black 65+ | 0.8 | 8 | 1.1 | 12 |
| Hispanic 18-29 | 14.5 | 157 | 8.1 | 88 |
| Hispanic 30-49 | 12.8 | 138 | 14.5 | 157 |
| Hispanic 50-64 | 4.9 | 53 | 6.6 | 71 |
| Hispanic 65+ | 0.4 | 4 | 1.9 | 20 |
| Gender and Age |  |  |  |  |
| Man 18-29 | 13.7 | 148 | 6.4 | 69 |
| Man 30-49 | 16.6 | 179 | 15.4 | 166 |
| Man 50-64 | 8.8 | 95 | 11.0 | 119 |
| Man 65+ | 3.3 | 36 | 7.0 | 76 |
| Woman 18-29 | 20.3 | 220 | 12.5 | 135 |
| Woman 30-49 | 21.0 | 227 | 26.2 | 283 |
| Woman 50-64 | 11.4 | 123 | 14.9 | 161 |
| Woman 65+ | 4.8 | 52 | 6.6 | 71 |
| Total |  | 1,080 |  | 1,080 |

| S1 Table 2. Proportion Harassed by Gender, Race, and Age | |
| --- | --- |
| Gender |  |
| Man | 0.73 |
| Woman | 0.77 |
| Race-Ethnicity |  |
| White | 0.70 |
| Black | 0.82 |
| Hispanic | 0.79 |
| Age |  |
| 18-29 | 0.85 |
| 30-49 | 0.72 |
| 50-64 | 0.72 |
| 65+ | 0.56 |

Proportion of Respondents Harassed and Comparisons of Gig and Conventional Workers.

Discarding our ordinal measure of how *often* respondents were harassed to focus instead on the proportion who were ever harassed while doing gig work would make the patterns a bit easier to grasp, but it would also discard information and probably underestimate group differences. Also, it would not produce estimates of harassment that can be compared with the proportions reported in studies of harassment in conventional jobs [1–4]. Even if we were to focus exclusively on sexual harassment, the single-question measure of sexual harassment would probably produce different estimates of sexual harassment than approaches that ask about a range of specific sexual harassment behaviors. Furthermore, the measures we use ask if respondents have been harassed since they started doing gig work rather than about harassment in the last 12 months. Even if the time frames were the same, it is difficult to compare gig and conventional work because people tend to spend relatively few hours doing gig work. The cumulative time spent at risk of harassment in gig work is thus likely to be far less over any set period of time. Comparisons of harassment in conventional and gig jobs in the year Pew collected the data [5] are further complicated by the fact that many people were still doing conventional jobs remotely due to the pandemic and thus were probably at a lower risk of harassment in conventional jobs than normal [4]. For all these reasons, we rely primarily on our ordinal harassment index rather than on proportions, and we focus on comparisons among gig workers. Comparisons of gig and conventional workers are not possible with these data anyway, because there were no questions about harassment in conventional jobs.

1.	Graf N. Sexual harassment at work in the era of #MeToo. Pew Research Center; 2018. Available: https://www.pewresearch.org/wp-content/uploads/sites/20/2018/04/Pew-Research-Center-Sexual-Harassment-Report-April-2018-FINAL.pdf

2.	Maestas N, Mullen K, Powell D, Von Wachter T, Wenger J. Working conditions in the United States: results of the 2015 American Working Conditions Survey. RAND Corporation; 2017. doi:10.7249/RR2014

3.	McLaughlin H, Uggen C, Blackstone A. The economic and career effects of sexual harassment on working women. Gender & Society. 2017;31: 333–358. doi:10.1177/0891243217704631

4.	Rosenfeld MJ. The surprising decline of workplace sexual harassment incidence in the U.S. federal workforce. Sociological Science. 2024;11: 934–964. doi:10.15195/v11.a34

5.	Anderson M, McClain C, Faverio M, Gelles-Watnick R. The state of gig work in 2021. Pew Research Center; 2021.
